# Supplementary material for: Expanding Possibilities for Foreign Gene Expression by Cucumber Green Mottle Mosaic Virus Genome-Based Bipartite Vector System
Source: Plants (Basel). 2024 May 19;13(10):1414. doi: 10.3390/plants13101414 (PMC11124972; doi:10.3390/plants13101414)
Supplement: Supplementary file 1 [file plants-13-01414-s001.zip › plants-2942528-supplementary.pdf]

Table S1. Details of the primers used in this research programme

Primer Name	Primer sequence (5'- 3') [a]	Location (ORF)	Nucleotide coordinate	Amplicon size (bp)
BM 486F	***CGGGATCC***GTTTTAATTTTTAAAATTAAAC	5′UTR	1-22	6400
BM 489R	***GCTCTAGA***TGGGCCCTTACCCAGG	3′UTR	6426-6442	
BM 962F	AGGCTACCACCTCGAAAGCTTAGGGAGGGCAATCTTATGTTGAAGC	CP+ NbPDS	6226-6248	227
BM 963R	ACCACCATCAGAAGACCCTCGAAAGGAGGGTTACCATCTAAAAAGG	UTR+NbPDS	6249-6272	
BM 1171F	GGTGATGATTCGCTGATCTACCTTCC	RdRp	4591- 4616	1415nt (in BP4)
BM 1172F	ATTGGGGCTCAGTTTAGGGTCGATGG	MT	802-827	768 nt (in DG1)
BM 1173R	GAAGATAGGCCTCAACACAGGACCG	CP	5981-6005	
BM 1178F	ATG GCT TAC AAT CCG ATC ACA CC	CP	5763-5785	486
BM 1179R	AGC TTT CGA GGT GGT AGC CTC	CP	6245-6225	
BM 1180F	AGCCGCGTTTTCGGTAGTCTGG	CP	6200-6221	197
BM 1269R	GCATCGAAAGCTCGTCAGGG	*NbPDS*	129-148	
BM 1265F	GCCAACACTTGTCACTACTTTCTC	GFP	183-206	230
BM 1266R	GATGTTTCCGTCCTCCTTGAAATC	GFP	397-420	
BM 849F	GGTATTGTGTTGGACTCGGG	*ACT1*	219-238	160
BM 850R	GCTGTGGTAGTGGATGAGTAAC	*ACT1*	361-382	
BM 96F	TGCTAAGCAAATCCCGTTTAT	RdRp	2943-2963	840
BM 1268R	GAATGCGCGGCCTTTCACAG	RdRp	3765-3784	
P1-F	CACCTCGAAAGCTTAGGGAGGGCAATCTTATGTTGA	NbPDS		Insertion of NbPDS gene within DG-1 constcruct at CP end
P2-R	ATCAGAAGACCCTCGAAAGGAGGGTTACCATCTAAAA	NbPDS		
P4	TGCCCTCCCTAAGCTTTCGAGGTGGTAGCCTCTGACCAGACTACCG	DG-1+NbPDS		
P5-R	CATCATCACCATCGACCCTAAACTG			Develoment of DG-1 construct from CGMMV genome based infectious construct
P6-F	GCTCTGGTTGACACAAGGATGCATTC			
P7	TCGATGGTGATGATGGCTCTGGTTGACACAAGGA			

**GGATCC* is the restriction site of BamH1, *TCTAGA* is the restriction site of XbaI. The primer set (BM 979F and BM 980R) is used for the deconstruction of CGMMV genome, whereas the primer set (BM-962 & BM-963R) is used for the insertion of the *NbPDS* gene into the DG-2 construct, RT-PCR based detection was carried out by using terminal primer set (BM-486F & BM-489R).

Table S2. The details about the binary vectors used in this study

Vector	Plant selection marker	Bacterial selection marker	Size (kb)	Reporter	Specification
pBM1 (Modified pCambia 2300)	Kan	Kan	9.2	Nil	Modified through the insertion of with 35S promoter and ribozyme sequence (RZ) and nos terminator
pBP4 (Symptomatic infectious cDNA clone of CGMMV)	Kan	Kan	15.6	Nil	Full-length CGMMV infectious cDNA was cloned into pBM1 binary vector with BamH1 and XbaI restriction sites
pBP7 (Asymptomatic infectious cDNA clone of CGMMV)	Kan	Kan	15.6	Nil	Artificially created through site-directed mutagenesis from symptomatic CGMMV (pBP4)
Modified pGreen II	Kan	Kan	3.4	LacZ	Modified through the insertion of with 35S promoter and ribozyme sequence (RZ) and NOS terminator

Table S3. RT-PCR based detection of DG-1 constructs infiltrated in the CGMMV infected *N. benthamiana* and cucurbits

Host	BP4[@] + DG1	BP7[$]+ DG-1	BP4	BP7	DG-1	Healthy[#] Control
N. benthamiana	18/18[*]	18/18	0/18	0/18	0/18	0/18
**Cucurbits**	0/18	0/18	0/18	0/18	0/18	0/18
Bottle Gourd	0/18	0/18	0/18	0/18	0/18	0/18
Water melon	0/18	0/18	0/18	0/18	0/18	0/18

*No of plants showing positive result/ total number of plants tested (in tri replicate)

@BP4 is the name of the symptomatic infectious clone of CGMMV

$BP7 is the name of the asymptomatic infectious clone of CGMMV

Healthy control represents the buffer (without agro-construct) infiltrated plant

Table S4. Trans-replication of DG2 and its GFP expression ability in the infiltrated leaves of CGMMV infected *N. benthamiana*

**Treatment**	**Detection of DG-2**		
	**No of plant showing RT-PCR positive for DG2 construct/ Total no of plant tested (%)**	**No of plant showing GFP expression/Total no of plant tested in Confocal microscopy (%)**	**Duration of GFP expression (DPI)**
BP4 + DG2	12/12(100)	6/12(50)	2-12
BP7 + DG2	12/12(100)	8/12(66.7)	2-15
BP4	0/6(0)	0/6(0)	-
BP7	0/6(0)	0/6(0)	-
DG2	0/6(0)	0/6(0)	-
Healthy Control	0/6(0)	0/6(0)	-

[1]The experiment was replicated twice. The DG-2 was agro- infiltrated into the infected *N. benthamiana* after 7DPI (days post infiltration) of the CGMMV symptomatic (BP4) and asymptomatic (BP7) clones. Healthy plants were infiltrated with only MES buffer solution. The RT-PCR was performed after 7DPI of DG-2 delivery. Confocal microscopy of the samples was performed at 2 days interval.

Table S5. Quantitative estimation DG-2 replicon in the infiltrated leaves of CGMMV infected *N. benthamiana*

Samples	Ct value	Log value (x) of Ct	Antilog value	Concentration (pg/µl)	Expected load of DG2 replicon (ng/g tissue)
2DPI	19.40	-6.26245	0.013026	0.134934	0.067467
4DPI	18.51	-5.67078	0.01963	0.195598	0.097799
6DPI	19.04	-6.08957	0.014684	0.15535	0.077675
8DPI	19.03	-6.08736	0.014707	0.156168	0.078084
10DPI	18.86	-5.80198	0.017924	0.167858	0.083929
12DPI	18.49	-5.70588	0.019158	0.194759	0.097379
15DPI	18.46	-5.57614	0.020961	0.197798	0.098899

*DPI indicates days post infiltration of DG2 at which the leaf samples were harvested. The threshold cycle (Ct) represents the average of three replicates. The log value of X was derived from the formula of the standard curve (Y= -1.655X+9.09, with R^2=0.99), where Y= Ct value. The original viral load was calculated from the antilog value of X with base value=2. The concentration of DG-2 RNA replicon was estimated in pg/µl, that was derived into the expected load (ng/g tissue).

Figures

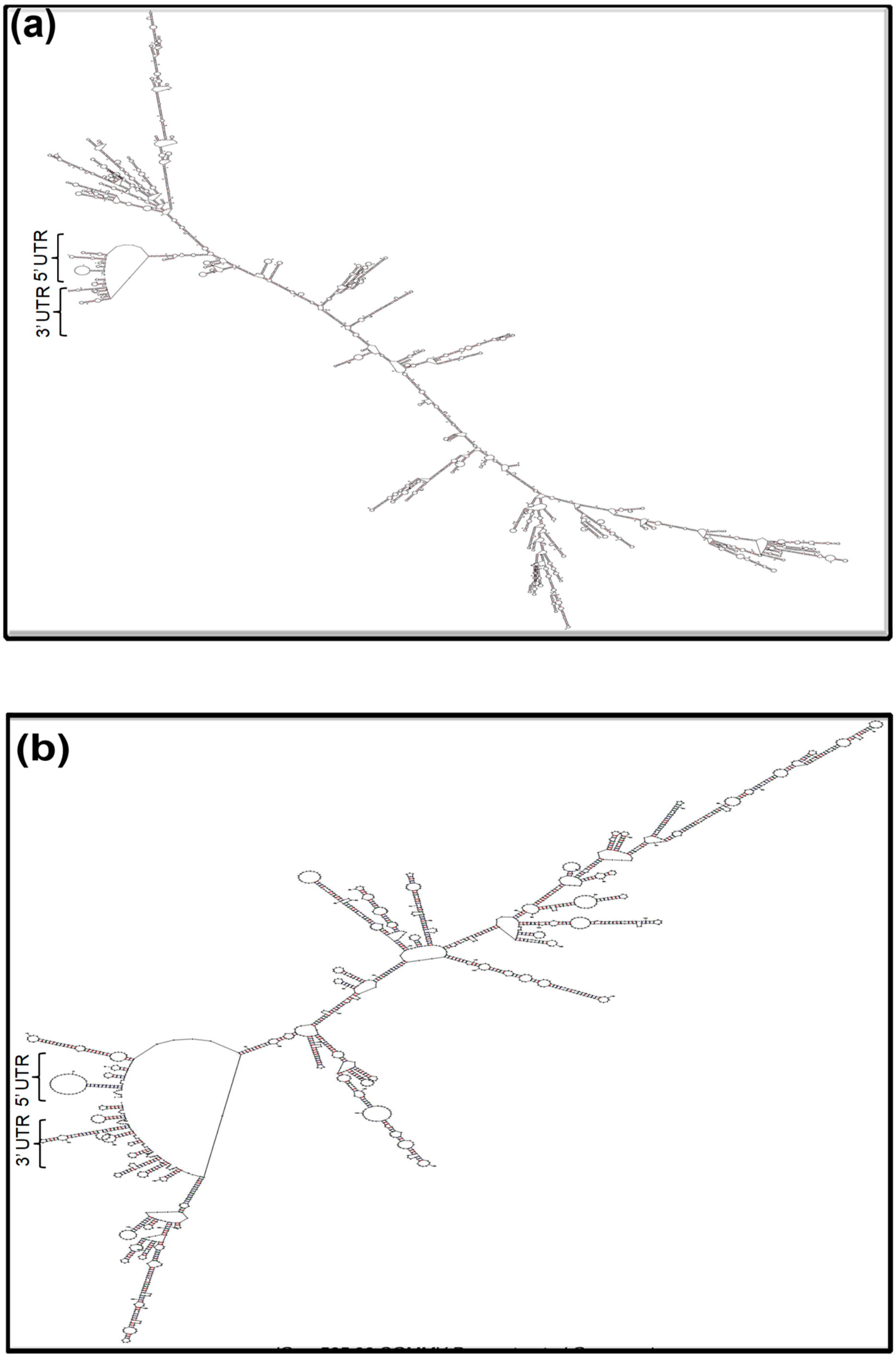

Figure S1. The RNA secondary structure of the deconstructed genome-1 (DG-1) of CGMMV is generated through the Mfold web-server. The structural conservation of 5' and 3' terminal end of DG-1 (~2.0 Kb) (b) depicts similarity with that of wild type genome of CGMMV (6.4 Kb) (a)

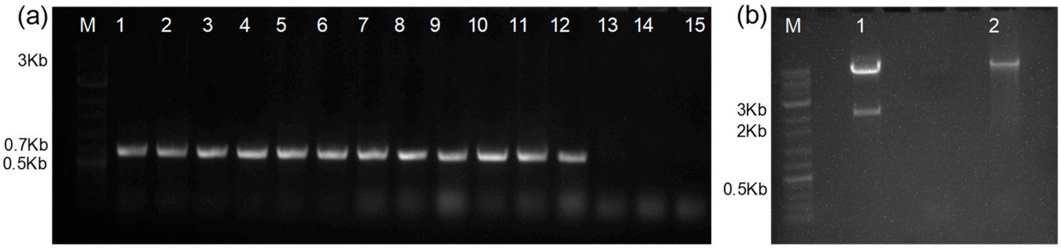

Figure S2. Development of deconstructed genome-1 using CGMMV genome based cDNA infectious clone (BP4) using LC-PCR technique. (a) The colony PCR based confirmation of the deconstructed genome using BM-1182F and BM-1173R primer which produces 768 bp amplicon for the DG-1 and 5204bp long amplicon for BP4. Colonies 1-12 are tested positive with rest (13-15) are negative. (b) The restriction digestion of the DG-1 plasmid construct with BamH1 and XbaI restriction enzyme. Lane-1 denotes the digested plasmid with ~ 2.0 Kb insert (DG-1) and Lane-2 is the uncut plasmid.

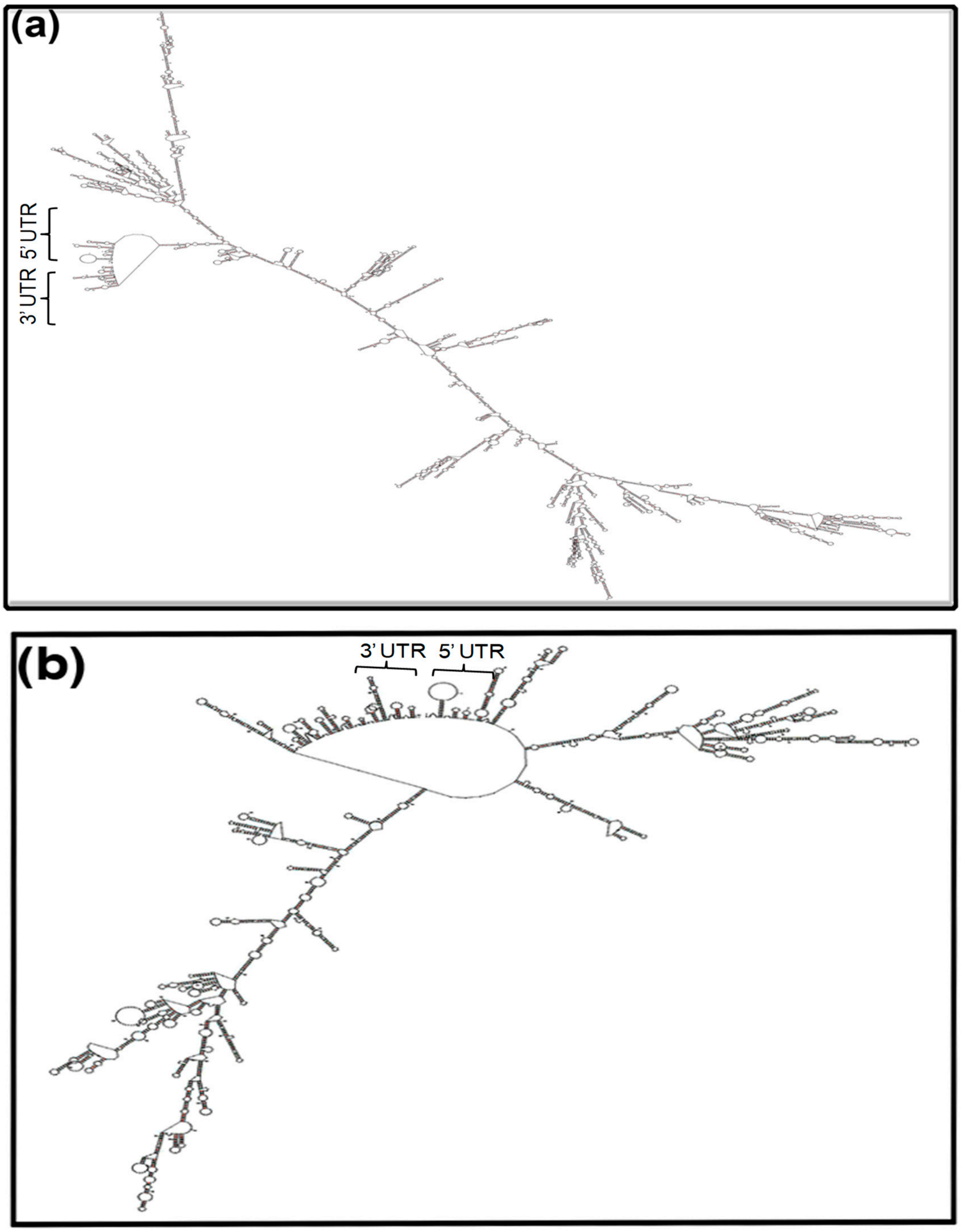

Figure S3. The RNA secondary structure of the deconstructed genome-2 (DG-2) of CGMMV is generated through the Mfold web-server. The structural conservation of 5' and 3' terminal end of DG-2 (2.9Kb) (b) depicts similarity with that of wild type genome of CGMMV (6.4Kb) (a).

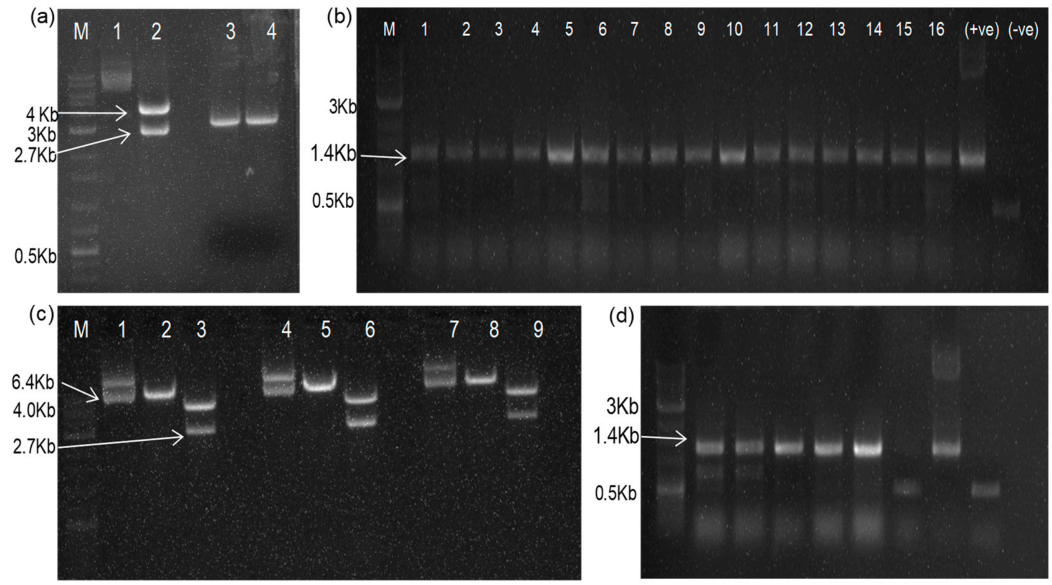

Figure S4. Cloning of deconstructed genome-2 synthetic construct from pUC57 to pGreenII 0029 vector. (a) The DG-2 was taken out from the pUC57(2.7kb) through restriction digestion restriction enzyme KpnI and SacI restriction enzyme. The digestion leads to the released 4062 bp long insert carrying 2x35s promoter, DG-2 genome (2961nt), and a ribozyme (Rz) site with *Nos* terminator. (b) Colony PCR for the detection of DG-2 constructs in pGreen II depicted to ensure the insertion of the entire synthetic gene cassette into pGreenII 0029 vector (4.6Kb) with KpnI and SacI restriction sites. The BM-1178F and BM-489R primer binding at 5' terminal of CP and 3' terminal end of 3'UTR of CGMMV, respectively, amplify the 1.4kb insert of DG-2 carrying eGFP (714 bp) fused to CP (486 nt) and 3'UTR (176 nt) against 662 nt long amplicons of wild CGMMV. (c) Restriction digestion of DG-2 from pGreenII backbone using BamH1 and HindIII released 2.786 Kb large internal fragments from the entire cassette and proved the stable insertion of DG-2 inside the pGreenII backbone. (d) Colony PCR based confirmation

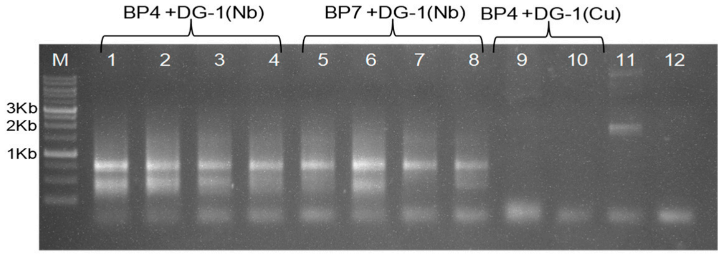

Figure S5. Detection of DG-1 constructs in *N. benthamiana* and cucurbits when co-infiltrated along with helper CGMMV constructs (BP4, and BP7). RT-PCR was performed by using BM-486F and BM-489R primer. DG-1 replicon was not detected when co-infiltrated with the 'helper' CGMMV. Lane **1**: Infiltrated (0) leaf (*N. benthamiana-1* infected with symptomatic BP4); **2**:1st systematic leaf (*N. benthamiana* -1infected with symptomatic BP4); **3**: Infiltrated (0) leaf (*N. benthamiana*-2 infected with symptomatic BP4); **4**:1st systematic leaf (*N. benthamiana* 2 infected with symptomatic BP4); **5**: Infiltrated (0) leaf (*N. benthamiana* -1 infected with asymptomatic BP7); **6**: 1st systematic leaf (*N. benthamiana* -1 infected with the asymptomatic clone); **7**: Infiltrated (0) leaf (*N. benthamiana* -2 infected with asymptomatic BP7 clone); **8**: 1st systematic leaf (*N. benthamiana* -2 infected with asymptomatic clone BP7); **9**: 1st systemic leaf (Cucumber-1); **10**: 1st systemic leaf (Cucumber-2); **11**: positive control (DG-1 plasmid); **12**: negative control.

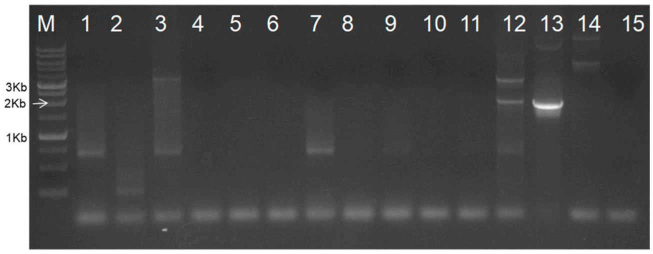

Figure S6. RT-PCR detection of DG-1 replicon in CGMMV infected cucurbits in comparison with CGMMV infected *N. benthamiana* using BM-486 F & BM-489R. DG-1 replicon was not detected in lane 1 to11, despite its infiltration in the various CGMMV infected cucurbits. Lane 1 to 3 represents the CGMMV-infected cucumber plants with its healthy control in lane 4; Lane 5-7 represents the CGMMV infected bottle gourd plants with its healthy control in lane 8; Lane 9-11 represents the CGMMV infected watermelon plants. The DG-1 replicon was only detected in lane 12 depicting the CGMMV infected *N. benthamiana*. Lane 13 is DG-1 plasmid control; lane 14 is the BP4 plasmid control, and lane 15 is the negative (reagent) control.

.

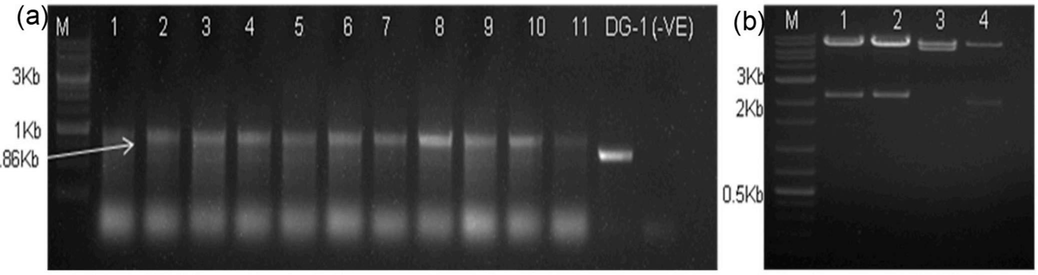

Figure S7. Insertion of partial *NbPDS* gene into the DG-1 construct. (a) The colony PCR based confirmation of the deconstructed genome using BM-1178F and BM-489R primer which produces 862 bp amplicon for the DG(PDS)-1 and 662bp amplicon for DG-1. Colonies 1-11 are tested positive. (b) The restriction digestion of the DG-PDS1 plasmid construct with BamH1 and XbaI restriction enzyme. Lane 1, and 2 denote the digested plasmid with~2.2 Kb insert, lane 3 is the digested BP4 plasmid with 6.4Kb insert, and lane 4 is the digested DG-1 plasmid with 2.0Kb insert.

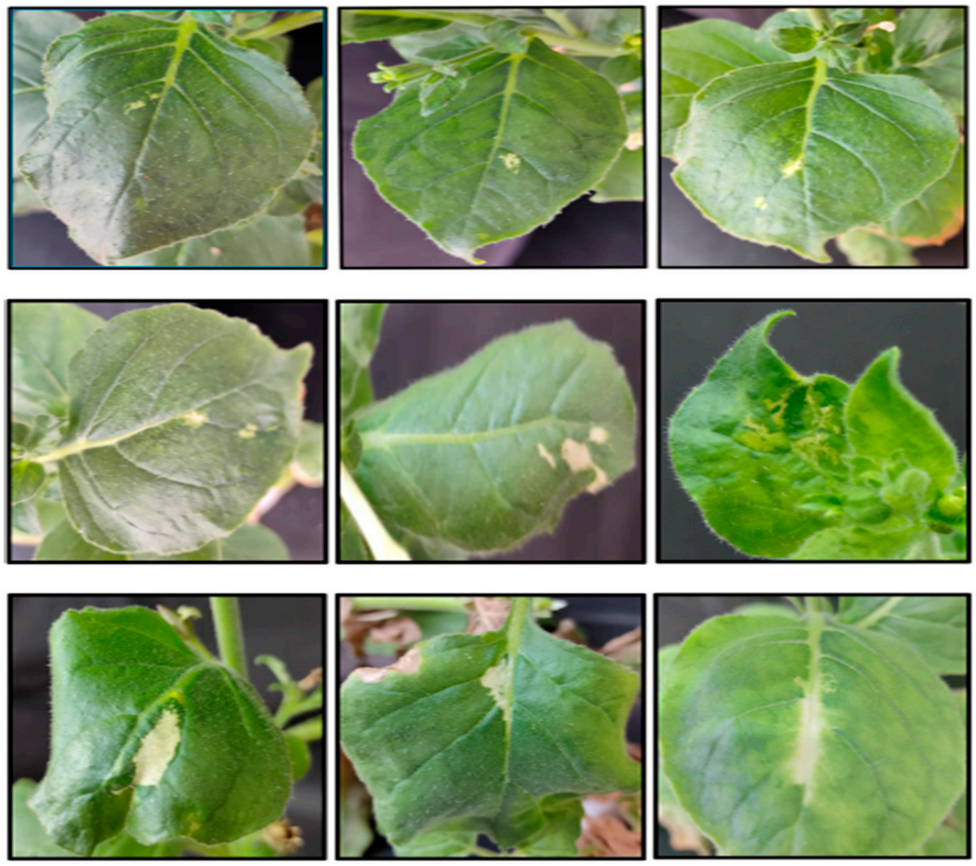

Figure S8. The evidence of photobleaching symptoms on the different leaves of *N. benthamiana* produced by DG(PDS)-1. The symptoms are scattered and observed on the few leaves only.

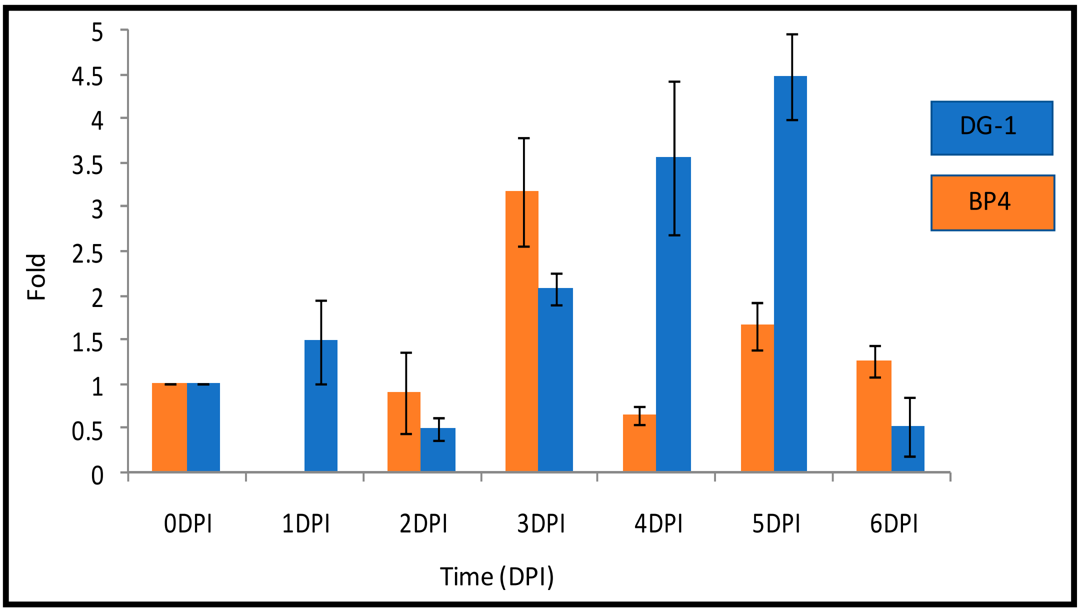

Figure S9. The quantitative presentation showed the replication pattern of DG(PDS)-1 replicon and its helper CGMMV (BP4) in *N. benthamiana* over the 0-6 DPI temporal scale. The test was performed using RNA samples extracted from infiltrated leaves of 10 different plants. The comparative analysis of fold change in the concentration of DG-1 replicon and helper CGMMV was calculated with reference to their concentration at 0DPI of DG(PDS)-1 in the CGMMV infected plant. The actin gene of *N. benthamiana* was used as the reference.

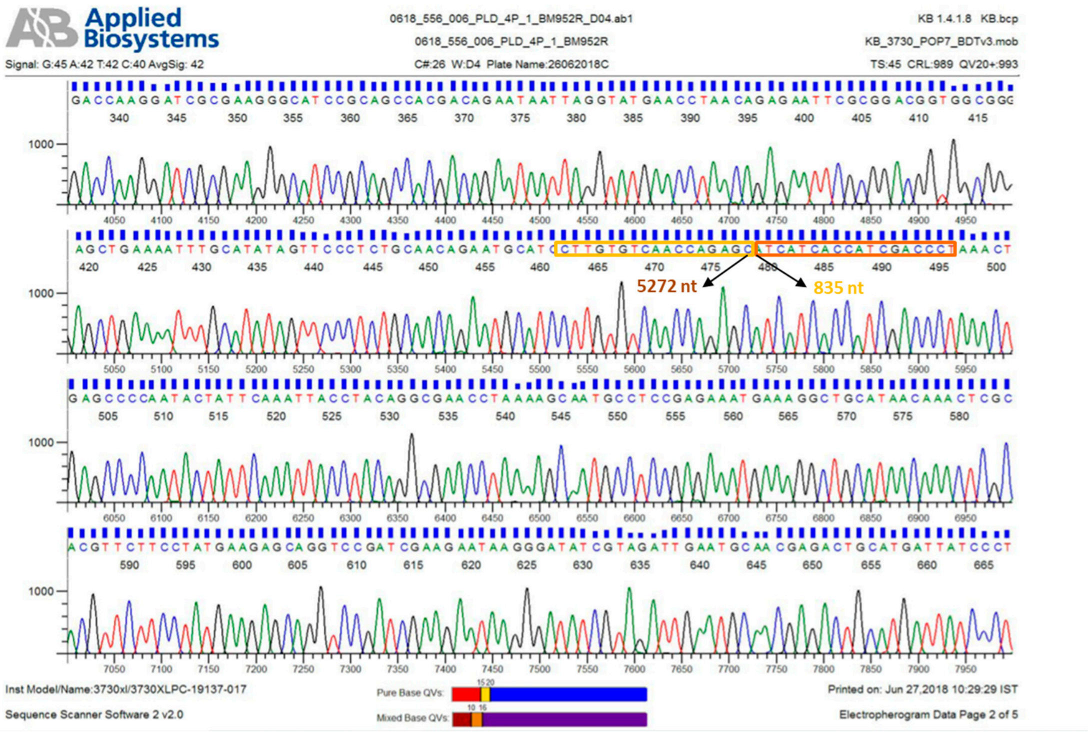

Figure S10: The DNA sequence-based confirmation of deconstructed genome, DG-1 generated through the deletion of RNA-dependent RNA polymerase (RdRP) and movement protein (MP) genes from the CGMMV genome by LC-PCR. The red box indicates the start position (834nt) of RdRp deletion and the yellow box indicates the end position (5271 nt) of MP deletion. The black arrow shows the deleted genomic part.

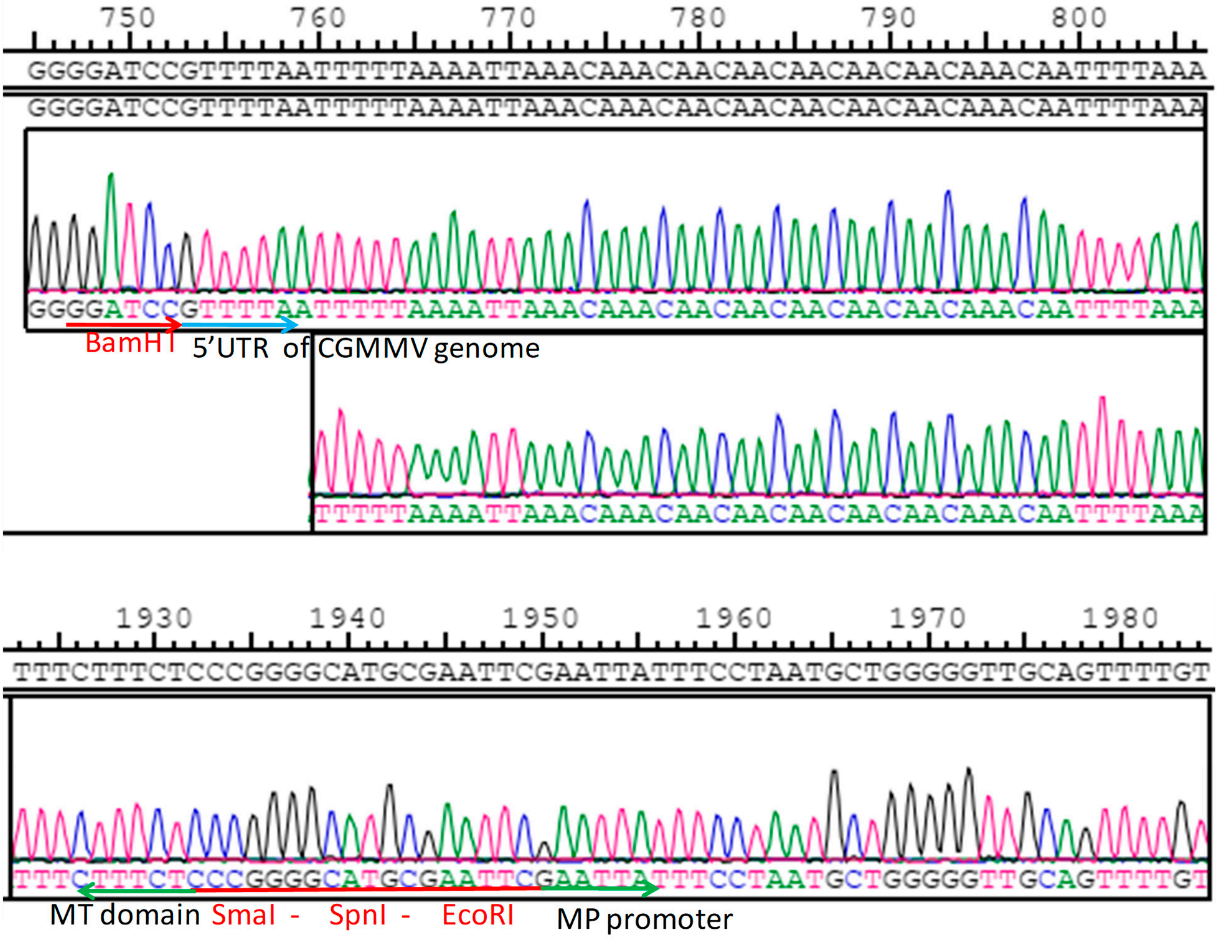

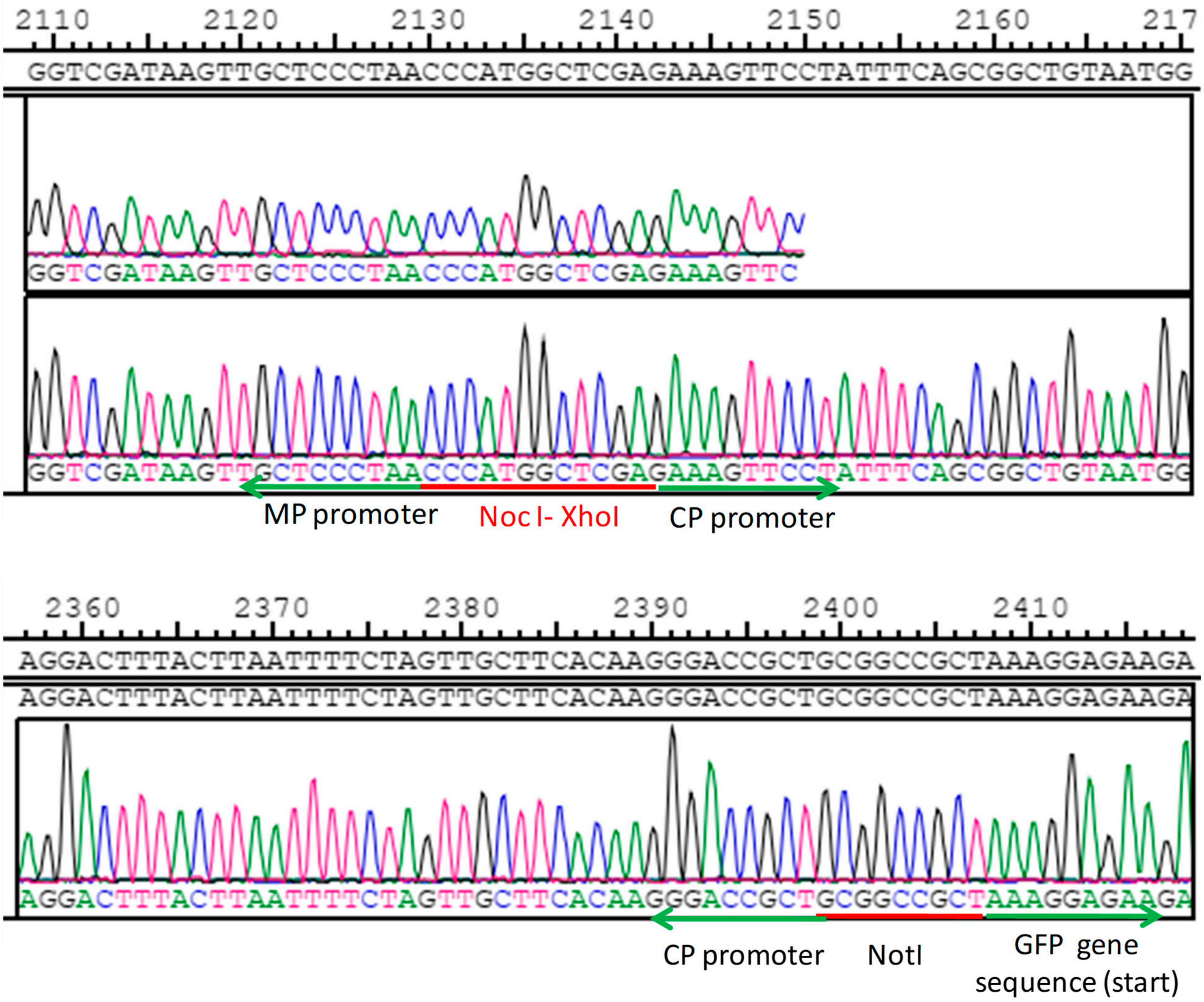
2110 2120 2130 2140 2150 2160 217
GGTCGATAAGTTGCTCCCTAACCCATGGCTCGAGAAAGTTCCTATTTCAGCGGCTGTAATGG
GGTCGATAAGTTGCTCCCTAACCCATGGCTCGAGAAAGTTC
GGTCGATAAGTTGCTCCCTAACCCATGGCTCGAGAAAGTTCCTATTTCAGCGGCTGTAATGG
MP promoter
Noc I- XhoI
CP promoter
2360 2370 2380 2390 2400 2410
AGGACTTTACTTAATTTTCTAGTTGCTTCACAAGGGACCGCTGCGGCCGCTAAAGGAGAAGA
AGGACTTTACTTAATTTTCTAGTTGCTTCACAAGGGACCGCTGCGGCCGCTAAAGGAGAAGA
AGGACTTTACTTAATTTTCTAGTTGCTTCACAAGGGACCGCTGCGGCCGCTAAAGGAGAAGA
CP promoter
NotI
GFP gene
sequence (start)

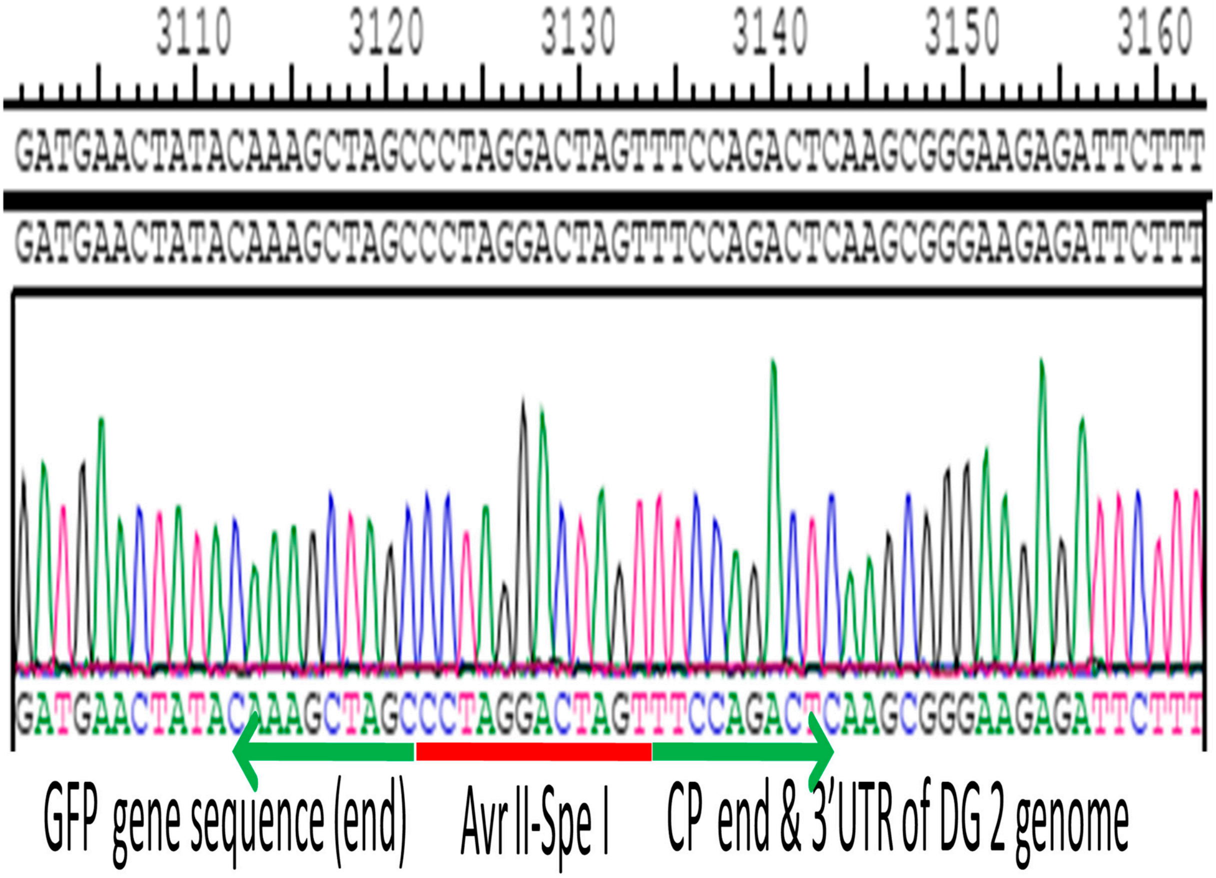

Figure S11. Sequence information of DG2 with multiple MCS (restriction sites) and GFP protein sequence.

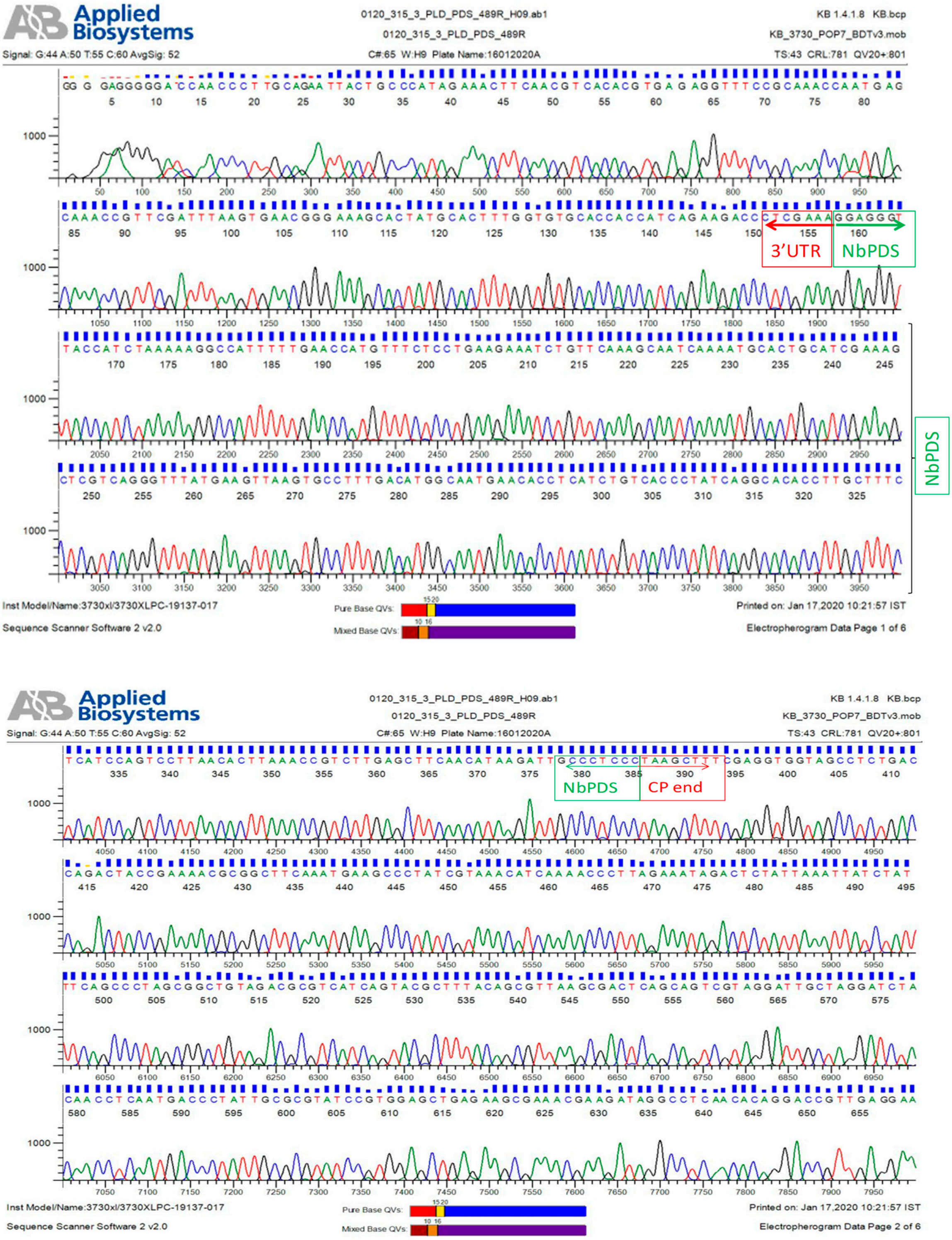

Figure S12. Sequence information of pDG(PDS)-1 Construct. The partial sequence (227nt) of NbPDS gene (EU165355) was inserted at the end of CP ORF of DG-1 genome confirmed by sequencing the constructs pDG(NbPDS)-1 by the reverse primer
